# A Cellulose Paper-Based Fluorescent Lateral Flow Immunoassay for the Quantitative Detection of Cardiac Troponin I

**DOI:** 10.3390/bios11020049

**Published:** 2021-02-14

**Authors:** Satheesh Natarajan, Joseph Jayaraj, Duarte Miguel F. Prazeres

**Affiliations:** 1 Healthcare Technology Innovation Centre, Indian Institute of Technology, Madras, Chennai, Tamil Nadu 600113, India; satheesh@htic.iitm.ac.in (S.N.); jayaraj@htic.iitm.ac.in (J.J.); 2 Department of Electrical Engineering, Indian Institute of Technology, Chennai, Tamil Nadu 600113, India; 3 IBB—Institute for Bioengineering and Biosciences, Department of Bioengineering, Instituto Superior Técnico, Universidade de Lisboa, 1049-001 Lisboa, Portugal

**Keywords:** biomarker, carbon nanofiber, cellulose, diagnostics, immunoassay, lateral flow assays, paper, point-of-care testing, troponin I

## Abstract

This paper presents a lateral flow assay (LFA) for the quantitative, fluorescence-based detection of the cardiac biomarker troponin I (cTnI) that features an analytical strip made of cellulose filter paper. The results show that the wicking and test time are comparable to those obtained with conventional nitrocellulose (NC)-based LFAs. Further, the cellulose paper provides an excellent background with no auto-fluorescence that is very adequate in detecting fluorescent lines. While fluorescence that was generated with cellulose strips was lower when compared to that generated in NC strips, signals could be improved by layering carbon nanofibers (CNF) on the cellulose. A nonlinear behavior of the concentration–response relationship was observed for the LFA architectures with NC, cellulose, and cellulose-CNF in the 0 to 200 ng/mL cTnI concentration range. The measurements were consistent and characterized by coefficients of variation lower than 2.5%. Detection and quantitation limits that were in the range 1.28–1.40 ng/mL and 2.10–2.75 ng/mL were obtained for LFA with cellulose and cellulose CNF strips that are equivalent to the limits obtained with the standard NC LFA. Overall, we showed that commercially available filter paper can be used in the analytical strip of LFA.

## 1. Introduction

Lateral flow assays (LFA) are the dominant segment in the Point-Of-Care (POC) testing market. These portable devices are designed to perform diagnostics at the time and place of patient care [1,2,3]. The moving of screening, diagnosis, and monitoring testing from a laboratory setting to the field could be particularly useful in the context of (i) emergencies that require fast results for clinical and healthcare decision making, (ii) diagnosis of people in remote areas, (iii) regular monitoring of chronic patients, (iv) the testing of patients during primary-care appointments, and (v) auto-monitoring [1,2,3]. The target applications for LFA include infectious disease testing (e.g., influenza, HIV, hepatitis C); glucose, cholesterol, urine, haematology, pregnancy, and fertility monitoring; cardiac and tumor/cancer marker testing; coagulation and activated clotting time analysis; and, the control of drugs-of-abuse, among others [4]. The current SARS-CoV-2 coronavirus pandemic provides an excellent example of the advantages and complementarity of LFA diagnostics. In particular, and as the disease spreads, LFA tests are being extensively used to detect anti-viral antibodies (e.g., IgG, IgM) and, thus, determine who has been infected and what is the seroprevalence in the population [5,6]. Further, LFA that detect SARS-CoV-2 antigens have been developed for the rapid diagnosis of infection [7,8]. Apart from human diagnostics, testing at the point-of-contact with LFA is also being pursued in the veterinary, environmental, agro-food, forensics, and bio-defense areas [9,10].

**Figure 1:**
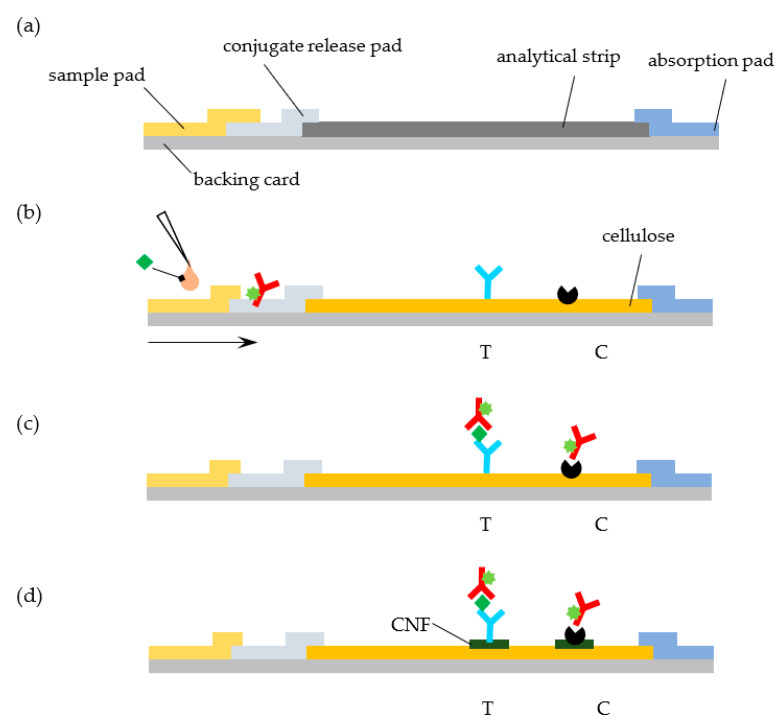
Lateral flow assays (LFA) with cellulose paper strip for detection of cardiac troponin I. (**a**) A standard LFA cartridge is made of overlapping materials mounted on a backing card: an analytical strip, usually made of nitrocellulose (NC), and sample, conjugate release and absorbent pads. (**b**) The LFA tested uses cellulose paper in the analytical strip instead of NC. Capture anti-cTnI IgG antibody (blue) and protein A (black) are adsorbed on the test (T) and control (C) lines, and Alexa Fluor-labeled anti-cTnI antibody (red) is impregnated in the conjugate pad. Upon addition of a sample of serum (~50 µL) spiked with cTnI (green lozenge), conjugates are released from the conjugate pad and form complexes with cTnI. (**c**) Anti-cTnI:cTnI complexes formed are captured by anti-cTnI antibodies in the test line. Unbound, residual complexes continue to move through the strip and are captured in the control line by protein A. (**d**) To improve detection, cellulose nanofibers (CNF) are layered over cellulose paper in the test and control line regions.

The LFA concept and the underlying technologies that are required for the manufacturing of the LFA hand-held cartridges at scale are well established [9,10,11]. At the heart of a conventional LFA, we find a series of overlapping rectangular strips of different components (sample pad, conjugate release pad, analytical strip, and absorbent pad) that are mounted on a backing card and combined with specific reagents for analyte recognition (Figure 1a). While changes to this architecture have been proposed (see, for example, Parolo et al., [12]), this simple design is predominant. Each material in the LFA accomplishes a specific function: (i) the backing card provides support, (ii) the sample pad receives the liquid sample, (iii) the release pad contains reagents that are required for the test, (iv) the analytical strip contains test and control areas where signals are generated and detected, and (v) the absorbent pad acts as a sink to receive the liquid that runs through the LFA [13,14].

Porous nitrocellulose (NC) membranes are the material of choice for the analytical strip in most cases [13,14]. NC is prepared by incorporating nitro groups in the glucose units of cellulose chains via nitration. This NC polymer can then be cast in the form of membranes with thicknesses of the order 100–150 µm by controlling its precipitation from a solvent system. The resulting material strongly interacts with proteins via hydrophobic and hydrogen bonds interactions, with binding capacities of more than 100 μg of IgG per cm^2^ [14]. This is one of the characteristics that justify the popularity of NC as a substrate for molecular detection. While NC is hydrophobic, it is rendered hydrophilic during casting by adding surfactants [14]. This confers it the ability to move fluids by capillarity that is critical for LFA. Other key characteristics of NC that make it ubiquitous in LFA include high brightness, wide availability at low cost, and with a range of porosities and pore sizes (3–20 µm) that are compatible with capillary migration, and ease of handling [13]. Nevertheless, some authors have pointed out that NC is not necessarily the best matrix for an LFA. For example, the inability to control the orientation of most proteins following adsorption usually translates into a loss of recognition activity [13] and, hence, in the need to use excess protein. Other notable shortcomings include, for example, changes and inconsistencies in the flow rates over time, lot-to-lot variability, flammable nature, sensitivity to humidity, and an inherent brittleness [13,14].

Cellulose constitutes an attractive and popular material for biosensors and LFA [15]. It is often found in the sample and absorbent pads due to its absorptive capacity, low cost, tensile strength, wide availability, and suitability for rapid roll-to-roll manufacture. However, few reports describe the use of cellulose in the analytical strip of LFA. This is somewhat surprising, given the widespread use of cellulose in many analytical (bio) chemistry applications and the fact that cellulose strips could cost one order of magnitude less than NC strips. For example, the increasing popularity of microfluidic paper-based devices provides a clear demonstration that cellulose could potentially be used in the analytical strip of LFA [16,17,18,19]. In one very comprehensive study, Lappalainen et al. investigated the adequateness of paper that is derived from different pulps as a material for the analytical strip of LFA by examining properties, like brightness, wet strength, and lateral flow speed [20]. The authors demonstrated that a functioning hemoglobin LFA could be set up by using analytical strips made of 80 g/m^2^ paper derived from unbeaten, bleached eucalyptus pulp, and modified with a resin [20]. In a follow up study, the same group successfully implemented a direct sandwich assay for the detection of morphine in an LFA. where the NC, analytical membrane was substituted by paper manufactured in the house [21].

A couple more recent studies also demonstrate how LFA sensitivity to protein and nucleic detection can be enhanced by layering cellulose nanofibers (CNF) on the test line regions of NC strips [22,23]. The core idea is to promote the penetration of CNF into the top pores of NC and, thus, contribute to increase the amount of capture antibodies close to the surface of the strips. As a result, the density of selectively bound gold nanoparticles in the top part of test lines increases, enhancing the LFA sensitivity by 36.6% in the case of the detection of human IgG [22] and by 20-fold in the case of the detection of *Staphylococcus aureus* nucleic acid [23].

The goal of this work is to test whether the standard NC analytical strip in a LFA can be replaced by a commercially available cellulose paper strip without a significant loss of performance. We further study the effect of layering CNF on the test line regions of the cellulose strip in the signals generated (Figure 1d). As a model system, we consider the quantitative detection of cardiac troponin I (cTnI), a biomarker of myocardial cell damage, by a fluorescent LFA [24]. The system relies on the recognition of the target cTnI molecule by fluorescently labelled anti-cTnI detection antibodies and subsequent capture of the complex at the test line by an anti-cTnI capturing antibody (Figure 1b,c). The fluorescent signal that is generated at the LFA lines is then quantified while using a portable immunoanalyzer [25,26].

## 2. Materials and Methods

### 2.1. Materials

The cellulose paper strip (chromatography paper Whatman N.1), sample pad (CF4), conjugate pad (Fusion 5), absorbent pad (CF6), and Sephadex G20 were procured from Cytiva (Bangalore, India). The NC membrane (Hi-Flow Plus HF180) was purchased from Merck Millipore (Burlington, MA, USA). The mouse anti-cTnI capturing antibody (clone 4C2 for cTnI), the mouse anti-cTnI detecting antibody (clone 19C7 for cTnI), recombinant troponin I, protein A, and Alexa Fluor 647 were from Abcam (Cambridge, UK). PBS (137 mM NaCl, 2.7 mM KCl, 10 mM Na_2_HPO_4_, 1.8 mM KH_2_PO_4_) and PB (75.4 mM Na_2_HPO_4_-7H_2_O, 24.6 mM NaH_2_PO_4_H_2_O) buffers, NaOH, NaHCO_3_, NaN3, BSA, and Tween-20, N-methylmorpholine N-oxide (NMMO) were purchased from Sigma–Aldrich (St. Louis, MO, USA). CNF (ref. NG01NC0201, 10–20 nm width, 2–3 µm length) were bought from Nanografi Nano Teknoloji (Ankara, Turkey).

An Easy Printer Model LPM-02 from MDI-Advanced Microdevices Pvt. Ltd. (Ambala, India) was used to dispense lines over the analytical strip. The fluorescence signals that were generated at the LFA test and control lines were evaluated using ImageQuant (Figure S1 (Supplementary Materials)), a portable immunoanalyzer developed and designed at the Healthcare Technology Innovation Center (IIT, Madras, India) [25,26]. This instrument relies on a laser based confocal optics system to capture the fluorescence of the test and control lines of the LFA strips. The captured images are analyzed with LabVIEW™ software (National Instruments, Austin, TX, USA) to obtain signal data from test and control lines. The system uses intelligent image-analytics techniques that identify the reaction kinematics from a sequence of images, track the reaction progress and development of the test and control lines, identify the stabilization of the reaction, and calculate the test and control line areas and area ratios [25,26].

### 2.2. Antibody Labeling

The anti-cTnI detection antibody was labelled with the fluorescent dye Alexa Fluor 647 following the manufacturer’s instructions. The Alexa Fluor 647 dye binds to the primary amine group of proteins at high molar ratios without self-quenching, forming stable dye–protein conjugates. The detection antibody (1 mg/mL in PBS buffer) was incubated with a 20 molar excess of the dye at room temperature for one hour under constant stirring. The fluorescent conjugates were purified by size exclusion chromatography on a Sephadex G20 gel column run with PBS buffer. Following purification, the conjugates were mixed with 0.02% NaN_3_ and stored at −20 °C.

### 2.3. LFA Strip Assembly

The LFA strip was assembled by sequentially joining and partially overlapping four types of pads/materials: a sample pad (9.5 mm length) for the analyte application, a polyester fiber membrane (6 mm length) that holds the detection antibody-dye conjugate, a cellulose paper strip (27.8 mm length) to generate signals, and an absorbent pad (13.0 mm length). LFA with plain cellulose paper strips or with cellulose paper strips that were layered with CNF were also used. Layering of CNF was performed by dispensing a 0.5% (*w*/*w*) suspension of CNF in Milli Q water over the cellulose paper in the form of lines on the test and control region using the Easy Printer. The suspension was repeatedly dispensed on the same position to increase the concentration of CNF within cellulose pores (from one to six times, i.e., 0.5–2.5%, respectively). The cellulose strips were dried overnight at room temperature. The sample pad was soaked with PBS that was supplemented with 0.15% Tween-20, 1% sucrose, 0.5% BSA, and dried for one hour at RT. The conjugate pad was immersed in a 0.3 mg/mL solution of antibody-dye conjugate that was diluted in 100 mM PB buffer with 0.1% Triton, 0.1% BSA, 20% sucrose, and subsequently dried for one hour at 40 °C. The test zone of the LFA strips was observed by scanning electron microscopy (SEM) using a Quanta 200 SEM from FEI (Hillsboro, OR, USA) located at International Centre for Clean Water (ICCW), IIT Madras. Prior to analysis, the samples were coated with CNF that were dissolved in an NMMO solution using the Easy Printer instrument.

Capture anti-cTnI antibody and protein A were dispensed over NC, cellulose, or CNF-layered cellulose strips (1 µL/cm) at a rate of 0.2 mg/mL in 1 × PBS using the Easy Printer. The analytical strips were then kept at 37 °C for one hour. Finally, the pads and analytical strips were laminated with a partial overlapping of 2 mm and then cut with a width of 3.2 mm. The assembled LFA strips were kept at 4 °C until used.

### 2.4. Analysis of Troponin I Samples

Standard samples (0, 5, 25, 100, and 200 ng/mL) were prepared by mixing serum with solutions of cTnI that was prepared in 0.1 M PB buffer at a volumetric ratio of 1:49 *v*/*v*. For analysis, 50 μL of each standard were added to the LFA cartridge, which was then inserted into the ImageQuant analyzer. The run button was pressed and the process was monitored for about 15 min. The images of the test and control lines of the cellulose paper strip were captured and analyzed to quantify the fluorescence intensity of the generated signals. Assays were performed in triplicate for each sample.

All of the quantitative data were assessed with GraphPad Prism 6.0 (GraphPad Software, La Jolla, CA, USA). Fluorescence intensity data were used to calculate the pixel volume of the test, V_T_, and control, V_C_, lines, which correspond to the two-dimensional summation of all pixel intensities within each line [26]. The corresponding mean volume ratio, V_R_, defined as the ratio V_T_/V_C_, was plotted versus the cTnI concentration to generate calibration curves. The standard deviation, SD, across triplicates was used to calculate the coefficient of variation (CoV) according to CoV = SD/mean × 100%. Calibration curve data were fitted to a power function, as described in Supplementary Materials. The limits of detection (LOD) and quantitation (LOQ) were determined based on the residual standard deviation and slope of the calibration curves obtained [27] (see Supplementary Materials).

## 3. Results and Discussion

Sandwich type assays for cTnI have been implemented in LFA while using detection strategies that rely on fluorophores [24], quantum dots [28], gold nanoparticles [29], or Raman tags [30], to name a few. On all of these systems, as in most LFA devices, NC is used in the analytical strip. Here, we examine whether the commercially available Whatman N. 1 chromatographic paper can be used instead of NC in a LFA for cTnI detection.

The cTnI LFA system used here relies on the recognition of the target cTnI molecule by fluorescently labelled anti-cTnI detection antibodies. Anti-cTnI capture antibody and protein A are immobilized on the test and control lines of the strip and conjugates of Alexa Fluor and anti-cTnI detection antibody are impregnated in the conjugate pad, as illustrated in Figure 1b,c. Upon sample addition and migration through the conjugate pad, conjugates are released, and they bind to the target analyte. The formed complexes then move through the cellulose paper strip and are captured by the anti-cTnI antibody adsorbed in the test line. Unbound, residual complexes continue to move through the strip, and they are captured in the control line by the adsorbed protein A.

The experiments were performed using LFA that was assembled with three different materials as analytical strips: (i) cellulose-LFA, (ii) cellulose-CNF-LFA, and (iii) NC-LFA. Cellulose-LFA were assembled using 27.8 mm length strips of Whatman N. 1 paper, a cellulose based (>98%) porous material defined by 15 µm fibers that has a basis weight of 87 g/m^2^ (Figure S2 (Supplementary Materials)). This paper features pores with a size distribution centered around 5 μm and spanning the 1–19 μm range [31,32,33,34]. Aqueous solutions wick through Whatman N. 1 cellulose strips with flow times of the order of ~484 ± 69 s/4 cm, which are larger than the flow times that were obtained with typical NC membranes (e.g., 75–240 s/4 cm, [14]). Cellulose-CNF-LFA were assembled with paper strips that were pre-layered with CNF on the test zone. Control NC-LFA were also mounted while using conventional NC strips (Figure S2 (Supplementary Materials)).

In a typical experiment, a 50 μL sample is dispensed on the sample pad of the LFA cartridges. The cartridge is incubated and subsequently analyzed in the ImageQuant instrument. A 15-min incubation was found to be sufficient for the completion of the cellulose-LFA and cellulose-CNF-LFA, which is five minutes more than the time that is required to run the equivalent NC-LFA [24]. This is consistent with the difference in flow times for cellulose and NC reported above. No dimensional changes (curling, waving, and cockling) in the cellulose paper strip were observed following sample addition and completion of the test. Fluorescence images of the cellulose, cellulose-CNF, and NC strips in the LFA cartridges were then captured by the ImageQuant camera. Positive samples run in the cellulose and cellulose-CNF-LFA display well-defined and sharp fluorescent lines in the test and control zones that contrast significantly with the dark background that is provided by the cellulose (Figure S3 (Supplementary Materials)). This lack of background fluorescence indicates that Whatman paper is compatible with fluorescence detection. Following image processing by the instrument inbuilt software, the pixel volumes of the test (V_T_), and control (V_C_) lines were calculated and then used to determine the pixel volume ratio, V_R_ (see Shah et al. 2018 for details [26]).

**Figure 2:**
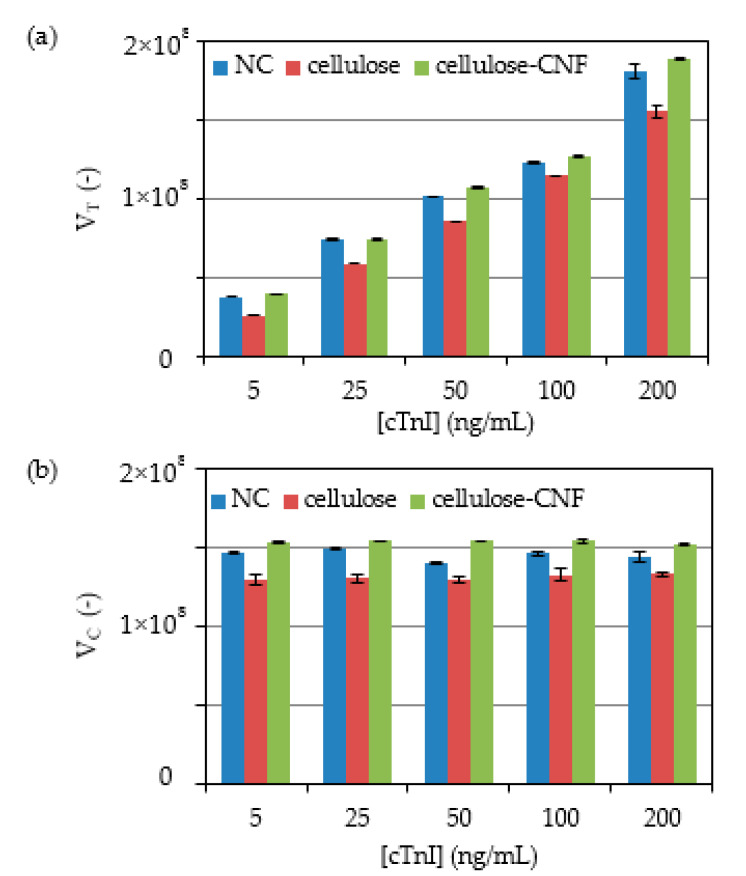
Effect of the concentration of samples containing cTnI on the immunofluorescence signals generated in the NC, cellulose and cellulose-CNF LFA. Pixel volumes of the (**a**) test and (**b**) control lines are shown. Experiments were run in triplicate.

Sets of experiments were performed with the three LFA types (NC, cellulose, cellulose-CNF) in triplicate while using serum samples that were spiked with known cTnI concentrations ranging from 0 to 200 ng/mL (Figure 2).

Fluorescence signals were obtained in the test lines of the LFA with a pixel volume, V_T_ that increases with cTnI concentration, as expected for the sandwich assay implemented (Figure 2a), whereas the pixel volume of the control line, V_C_, remained essentially constant (Figure 2b). A comparison of the signals that are generated across the cTnI concentration range shows that the replacement of NC for cellulose leads to a decrease in the fluorescence intensity of 7% to 31% and 7% to 13% for the test and control lines, respectively. These results are likely the consequence of paper properties that affect the distribution of capture and detection biomolecules over the test and control line volume. In particular, paper is thicker than NC (180 µm vs. 100 µm of the NC membrane used) and, thus, captured molecules will distribute over a larger volume of material, thus leading to lower densities at the line surface. Because the detection of fluorescence is mainly sensitive to complexes that are present on the surface of the strip, which is opaque, lower intensities are expected [29]. Nevertheless, we envisaged that fluorescence signals from cellulose strips could be improved by layering CNF on the test line regions of the paper strips, as described in the literature for the case of gold nanoparticle-based signals in NC strips [22,23]. Thus, LFA were prepared by depositing CNF generated from wood-derived fibrils with lengths in the micrometer and width in the nanometric range (Figure S4 (Supplementary Materials)), in the test and control regions of the strip. Photos of the strips with layered CNF obtained before and after running of tests provide evidence for the successful modification of CNF and confirm that the fibers are not washed away during the analysis (see Figure S5a–c (Supplementary Materials)). A comparison of the signals generated across the cTnI concentration range shows that the replacement of cellulose for cellulose-CNF results in a recovery of the intensity of signals generated at both the test and control lines, which even slightly surpassed the intensity of signals generated with NC strips (Figure 2).

**Figure 3:**
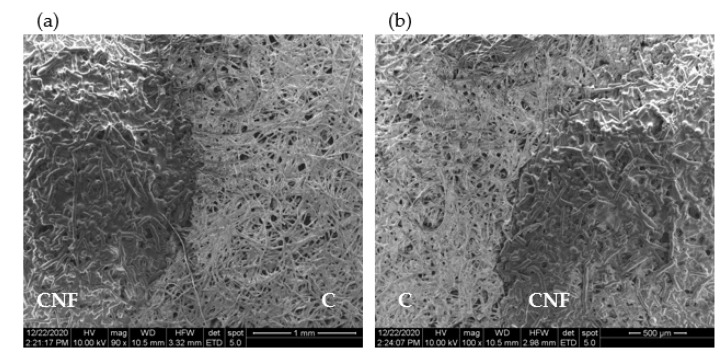
Scanning electron microscopy (SEM) analysis of cellulose strips with layered CNF at (**a**) 90×, and (**b**) 100× magnification. The boundary region between cellulose (marked C) and cellulose with layered CNF (marked CNF) is clearly visible.

A SEM observation of the test strips shows that the layering of the very thin and long CNF (Figure S4 (Supplementary Materials)) over cellulose significantly alters its microstructure (Figure 3 and Figure S5) by penetrating into and closing the pores of cellulose. The contrast between the CNF-layered cellulose and plain cellulose is quite evident in Figure 3a,b (also in Figure S5d,e (Supplementary Materials)), which captures the boundary region between the two zones at increasing magnification. It is also apparent from the figures that the deposition of CNF results in the covering of the cellulose microfibrils with a smoother mesh of material with a significantly lower porosity (Figure 3a). Given their dimensions, the numerous nanofibers deposited increase the cellulose surface area close to the top face of the strips. As a result, a larger amount of captured biomolecules will adsorb to the cellulose material close to the surface of the strips. This adsorption of antibodies to the cellulose fibers is likely to involve different types of interactions, e.g., hydrogen binding, van der Waals, electrostatic, and aromatic stacking interactions [35]. Subsequently, this translates into a higher density of fluorescence complexes at the surface and, hence, to an increase in the fluorescence signals.

The V_T_ and V_C_ data were used to calculate V_R_, a relative measured used as the response of the LFA devices (Tables S1–S3 (Supplementary Materials)). Triplicate V_R_ data were further used to compute the individual CoV, which were then averaged to yield the intra-assay CoV (see Tables S1–S3 (Supplementary Materials)). The values of average CoV of 0.48%, 2.43% and 0.69% were obtained for the NC-LFA, cellulose-LFA, and cellulose-CNF-LFA, respectively (Table 1). This provides a good indication that measurements of cTnI concentration in the devices are reliable and consistent.

The calibration curves were constructed next by plotting the replicate V_R_ data as a function of cTnI concentration for the three LFA types (NC, cellulose, cellulose-CNF). A nonlinear behavior of the concentration–response relationship was observed for the three different LFA architectures, which closely resembles a power-function response of the form:

(1) $$V_{R}=a{\left[\mathrm{cTnI}\right]}^{b}$$

where a and b are constants.

**Figure 4:**
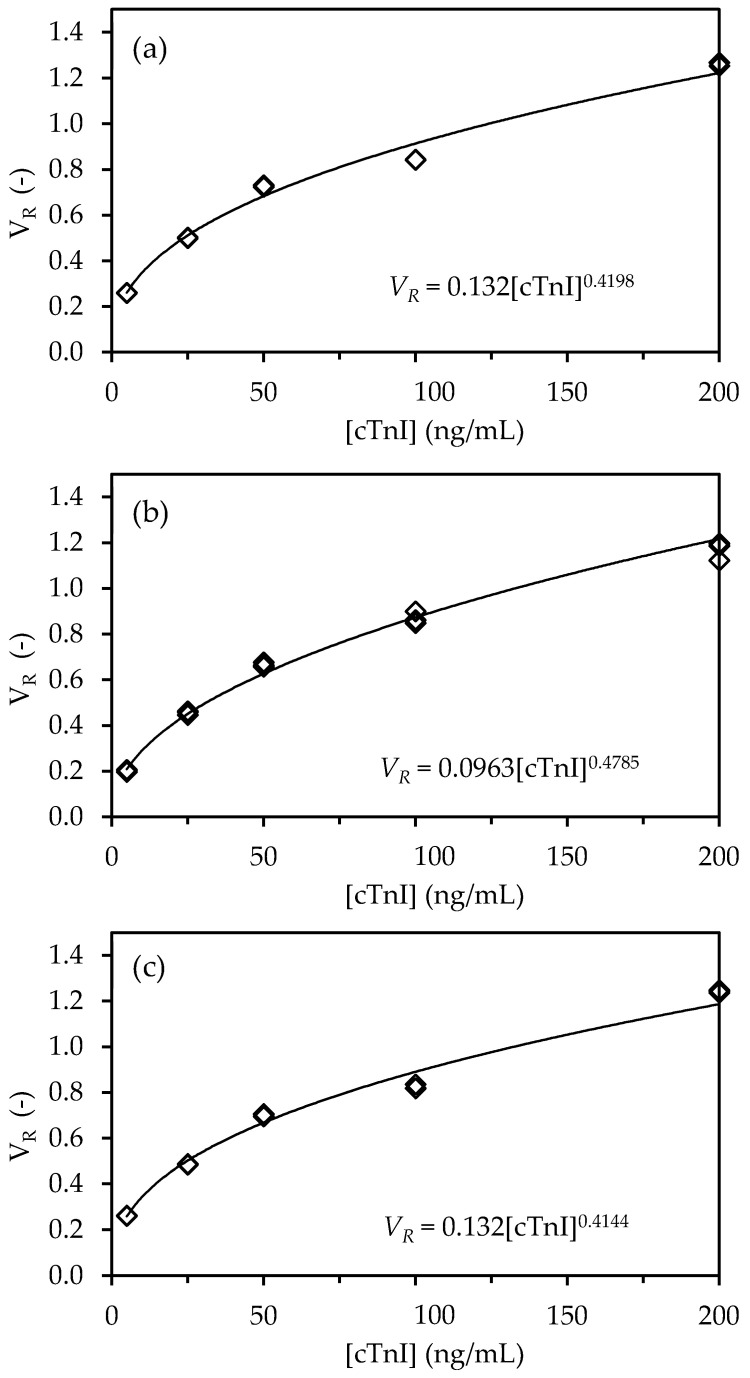
Calibration curves for the detection of cTnI with NC, cellulose and cellulose-CNF-LFA. The ratio of pixel volume of test line and control line (V_R_ = V_T_/V_C_) is plotted as a function of the concentration of cTnI ([cTnI]) for the (**a**) NC, (**b**) cellulose, and (**c**) cellulose-CNF-LFA. The experiments were run in triplicate and data were fitted with a power function of the type $V_{R}=a{\left[\mathrm{cTnI}\right]}^{b}$

Equation (1) was linearized and fitted to the experimental data using the regression function of Microsoft Excel (2010) to extract the values of parameters a and b for the three LFA architectures (see Figures S6–S8 (Supplementary Materials)). The regression statistics data (Table 1) showed that Equation (1) fitted the experimental data very well, as can be judged by Figure 4.

In healthy individuals, the cTnI concentration is recorded at 0.1–0.3 ng/mL. However, with the onset of AMI, the cTnI values increase considerably and remain high for several hours [36]. A cTnI cutoff level of 6 ng/mL at one hour has been defined as appropriate for an accurate and rapid exclusion and identification of patients with suspected AMI [35]. The LOD and LOQ of the three LFA types tested were determined based on the residual standard deviation and slope of the linearized calibration curves [27] (see S1 (Supplementary Materials)). The obtained values were equivalent across the three LFA types, with values in the range 1.28–1.40 ng/mL and 2.10–2.75 ng/mL obtained for the LOD and LOQ, respectively (Table 1). Thus, these limits are compatible with a use in the context of emergencies, where rapid triage is required to exclude/identify patients with AMI.

## 4. Conclusions

We show that commercially available cellulose filter paper can be used as the analytical strip in a LFA for the quantitative, fluorescence-based detection of cTnI while using a sandwich type assay. While the flow and test times were slightly larger than those obtained with conventional NC-based LFAs, analysis was complete within 15 min. Further, fluorescence signals from test and control lines could be read with an image-based analyzer and then used to produce quantitative results. CNF were successfully layered on the test line regions in order to increase the amount of capture antibodies close to the surface of the cellulose strips. This resulted in the generation of immunofluorescence signals that were identical to those that were obtained with NC strips. The concentration–response relationship in the 0 to 250 ng/mL cTnI concentration range displayed a non-linear behavior for the three LFA architectures that could be described by a power law function. Measurements of cTnI concentration across the devices were reliable and consistent, as judged by a CoV lower than 2.5%. The LFA with cellulose and cellulose CNF strips displayed detection and quantitation limits that were in the range 1.28–1.40 ng/mL and 2.10–2.75 ng/mL, which were equivalent to the limits obtained with the standard NC LFA. Overall, we provide evidence that commercially available filter paper possesses adequate characteristics to replace nitrocellulose as the material of choice for the analytical strip in LFA.

## Figures and Tables

**Table 1:** Fitting of the experimental response of the three LFA types (NC, cellulose, cellulose-CNF) to a power-function response of the type $V_{R}=a{\left[\mathrm{cTnI}\right]}^{b}$. The intra-assay CoV and the limits of detection and quantitation are also provided.

Parameter	NC	Cellulose	Cellulose-CNF
a	0.1320	0.0963	0.1320
B	0.4198	0.4785	0.4144
R	0.9958	0.9977	0.9957
CoV (%)	0.4771	2.430	0.6943
LOD (ng/mL)	1.39	1.28	1.40
LOQ (ng/mL)	2.73	2.10	2.75

## Data Availability

Not applicable.

## Supplementary Materials

The following are available online at https://www.mdpi.com/2079-6374/11/2/49/s1, Figure S1: Photo of ImageQuant, the Image-based Quantitative Immunoassay Analyzer developed at HITC and used to evaluate the fluorescence signals generated at the LFA test and control lines, Figure S2: Photo showing the assembled nitrocellulose (a), cellulose (b), and cellulose with deposited CNF strips before running the tests. In (c) the deposited CNF in the test and control zones is clearly visible, Figure S3: Representative black and white (a) and color (b) fluorescence images of cellulose paper strips with test (T) and control (C) lines in LFA cartridges as captured by the ImageQuant camera. The cTnI concentrations of samples run in the LFA are displayed next to each photo. (c) Profiles of the fluorescence intensity alongside the cellulose strips in (a). The gray value along the axis of the images of strips shown in (a) was measured using the Analyze/Plot profile tool of the Image J software (NIH, National Institutes of Health), Figure S4: TEM image of the NG01NC0201 (Nanografi Nano Teknoloji, Turkey) carbon nanofibers used, Figure S5: High-resolution photos of the cellulose strips (a), cellulose strips with deposited CNF before running the tests (b) and cellulose strips with deposited CNF after running the tests (c). SEM analysis of cellulose strips with layered CNF at (a) 200× and (b) 500× magnification. In (a) the boundary region between cellulose (marked C) and cellulose with layered CNF (marked CNF) is clearly visible, Figures S6–S8: Linear regression of the log V_R_ vs. log [cTnI] data for the NC, cellulose and cellulose-CNF LFA. Table S1–S3: Response of the NC, cellulose and cellulose-CNF-LFA to serum samples with different concentrations of cTnI and calculation of the corresponding intra-assay coefficient of variation.
