# Supplementary material for: A Cellulose Paper-Based Fluorescent Lateral Flow Immunoassay for the Quantitative Detection of Cardiac Troponin I
Source: Biosensors (Basel). 2021 Feb 14;11(2):49. doi: 10.3390/bios11020049 (PMC7918919; doi:10.3390/bios11020049)
Supplement: Supplementary file 1 [file biosensors-11-00049-s001.pdf]

biosensors MDPI

Supporting information for

A Cellulose Paper-Based Fluorescent Lateral Flow Immunoassay for the Quantitative Detection of Cardiac Troponin I

Satheesh Natarajan [1], Joseph Jayaraj [1,2] and Duarte Miguel F. Prazeres [3,*]

[1] Healthcare Technology Innovation Centre, Indian Institute of Technology, Madras, Chennai, Tamil Nadu 600113, India; satheesh@htic.iitm.ac.in (S.N.); jayaraj@htic.iitm.ac.in (J.J.)
[2] Department of Electrical Engineering, Indian Institute of Technology, Chennai, Tamil Nadu 600113, India
[3] iBB—Institute for Bioengineering and Biosciences, Department of Bioengineering, Instituto Superior Técnico, Universidade de Lisboa, 1049-001 Lisboa, Portugal
* Correspondence: miguelprazeres@tecnico.ulisboa.pt

This file includes:

Supplementary info S1

Supplementary Figures S1-S7

Supplementary Table S1-S3

S1. Fitting of Calibration Curves and determination of LOD and LOQ

A nonlinear behavior of the V_R *vs [cTnI]* calibration data was observed for the three different LFA architectures (analytical strip made of NC, cellulose or cellulose coated with CNF), which closely resembles a power-function response of the form:

$$V_R = a[cTnI]^b \qquad (S1)$$

where a and b are constants. This power function was then linearized by applying decimal logarithms to yield:

$$\log V_R = \log a + b\log[cTnI] \qquad (S2)$$

This equation was then fitted to the data as follows. First, the decimal logarithm of V_R and of *[cTnI]* was taken. Next a linear regression analysis of the log V_R *vs* log *[cTnI]* data was carried out using the regression function of Microsoft Excel (2010) to extract the values of parameters a and b for the three LFA architectures. The regression statistics data showed that equation S1 and S2 fitted the experimental data very well, as can be judged by Figures S4-S6. The limits of detection and quantitation were then computed using the standard deviation of response (σ) for the y intercept and the slope (b) of the log V_R *vs* log *[cTnI]* calibration curve according to [1]:

$$LOD' = 3.3\frac{\sigma}{b} \qquad (S3)$$

$$LOQ' = 10\frac{\sigma}{b} \qquad (S4)$$

The values of LOD´ and LOQ´ where then converted to the final LOD and LOQ according to:

$$LOD = 10^{LOD'} \qquad (S5)$$

$$LOQ = 10^{LOQ'} \qquad (S6)$$

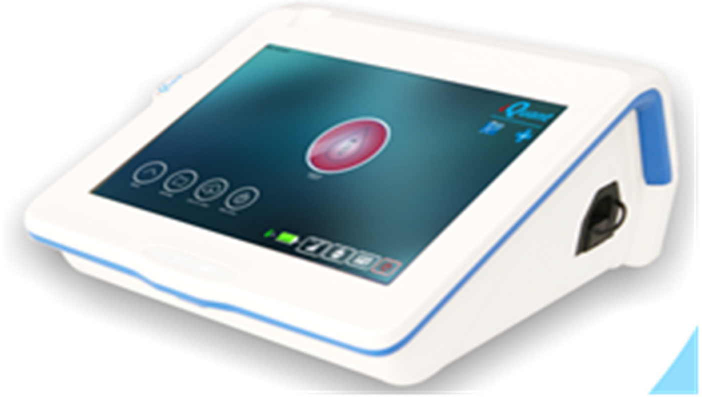

Figure S1. Photo of ImageQuant, the Image-based Quantitative Immunoassay Analyzer developed at HITC and used to evaluate the fluorescence signals generated at the LFA test and control lines.

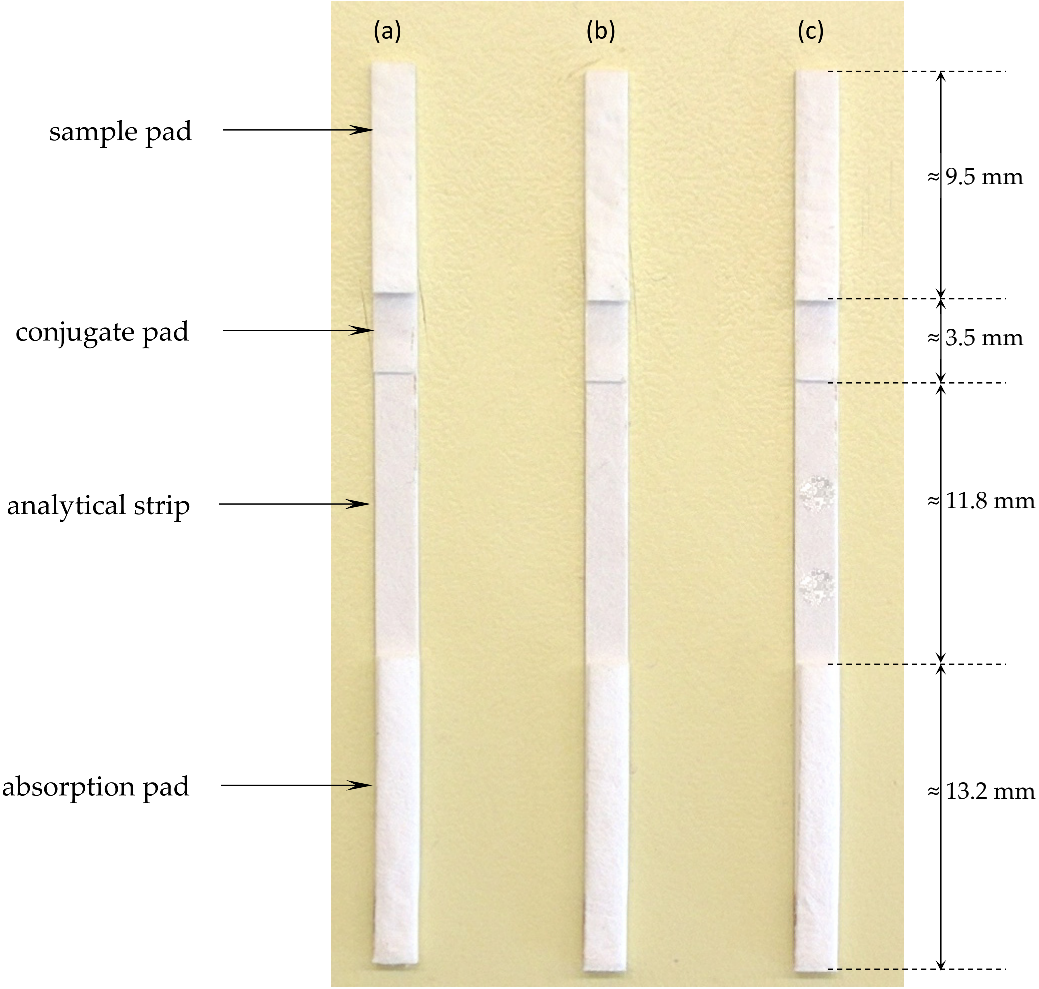

Figure S2. Photo showing the assembled nitrocellulose (**a**), cellulose (**b**), and cellulose with deposited CNF strips before running the tests. In (**c**) the deposited CNF in the test and control zones are clearly visible.

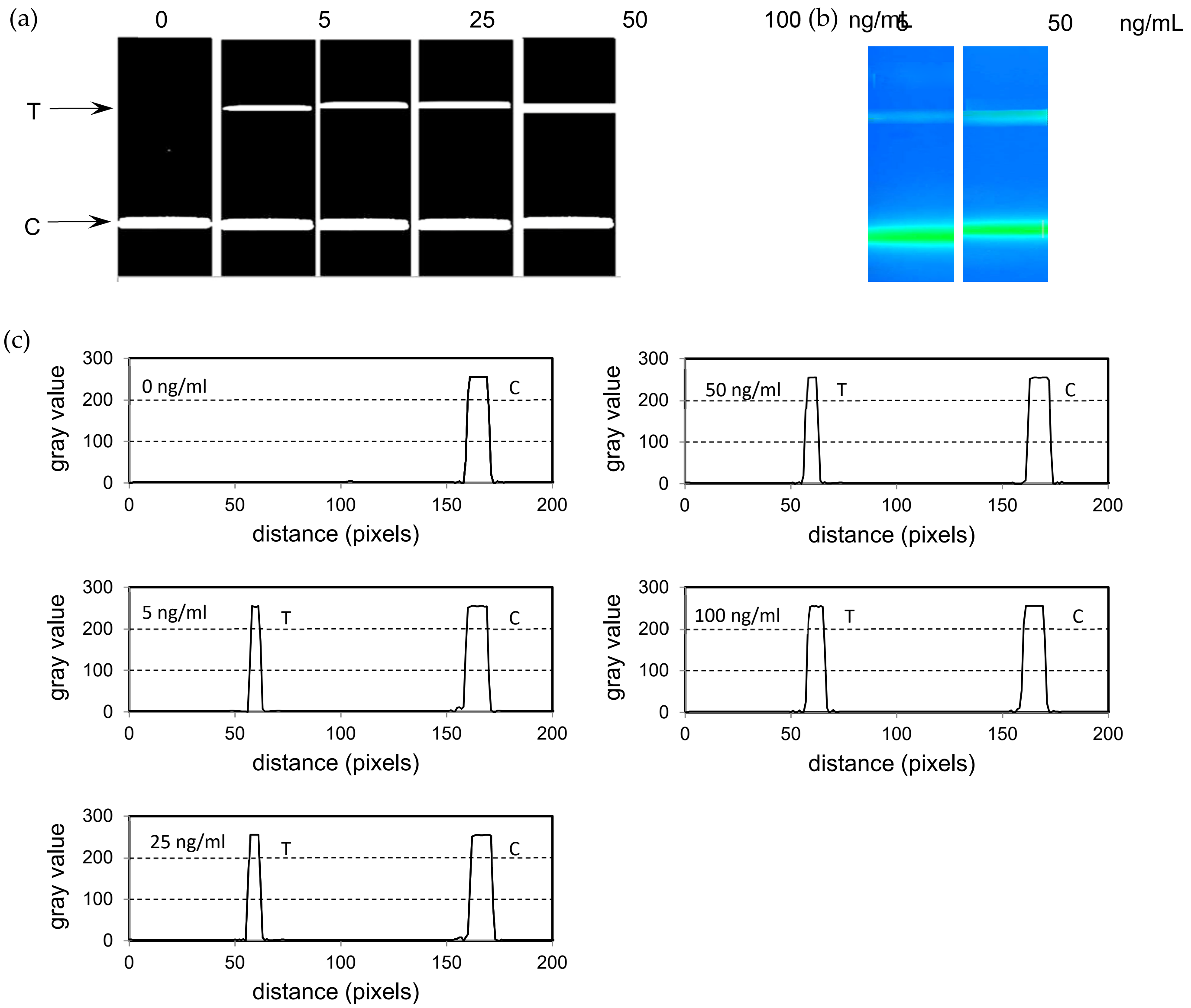

Figure S3. Representative black and white (**a**) and color (**b**) fluorescence images of test (T) and control (C) lines in the cellulose strips of LFA cartridges as captured by the ImageQuant camera. The cTnI concentrations of samples run in the LFA are displayed next to each photo. (**c**) Profiles of the fluorescence intensity alongside the cellulose strips in (a). The gray value along the axis of the images of strips shown in (a) was measured using the Analyze/Plot profile tool of the Image J software (NIH, National Institutes of Health).

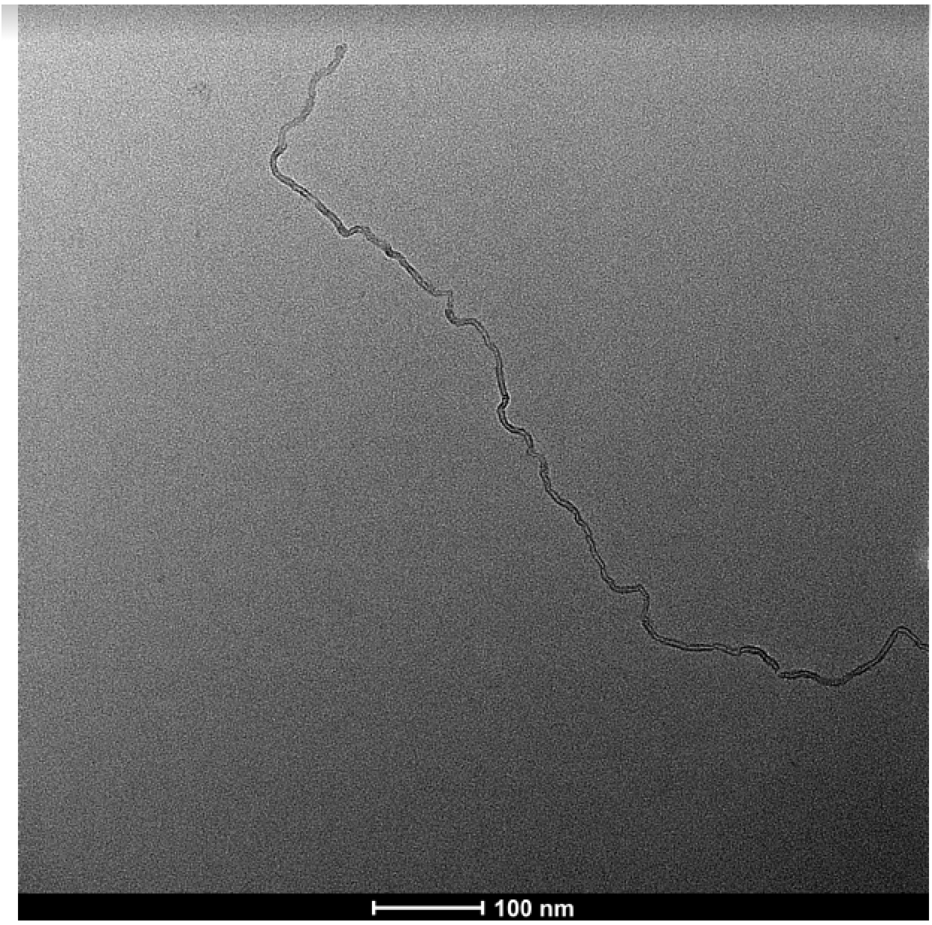

Figure S4. TEM image of the NG01NC0201 (Nanografi Nano Teknoloji, Turkey) carbon nanofibers used (reproduced with permission from Nanografi Nano Teknoloji, retrieved from https://nanografi.com/popular-products/cellulose-nanofiber-cellulose-nanofibril-nanofibrillated-cellulose-cnfs/, 2021).

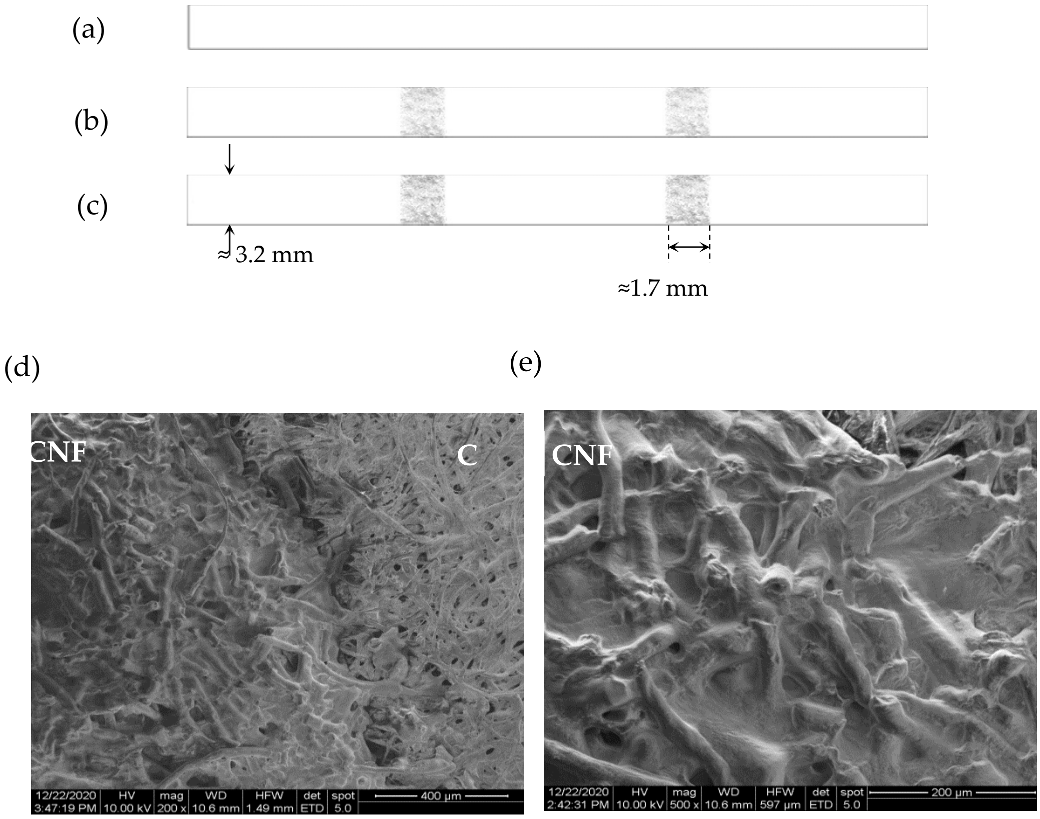

Figure S5. High-resolution photos of the cellulose strips (**a**), cellulose strips with deposited CNF before running the tests (**b**) and cellulose strips with deposited CNF after running the tests (**c**). The photos were edited to enhance the contrast between the CNF and cellulose. In (b) and (c) the boundary between cellulose and the deposited CNF, which appear darker, is clearly visible. SEM analysis of cellulose strips with layered CNF at (**d**) 200x and (**e**) 500x magnification. In (a) the boundary region between cellulose (marked C) and cellulose with layered CNF (marked CNF) is clearly visible.

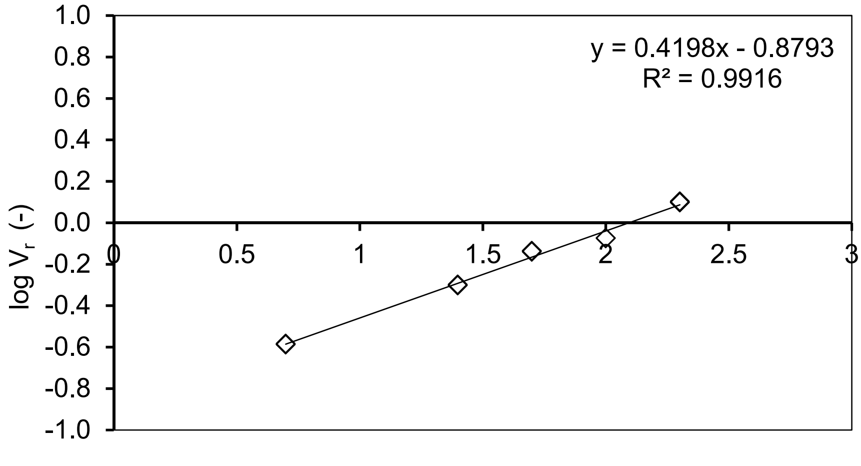

Slope	0.4198	LOD	1.39 ng/ml
Intercept	−0.8793	LOQ	2.73 ng/ml
Standard deviation (σ)	0.0183		

Figure S6. Linear regression of the log *VR vs* log *[cTnI]* data for the nitrocellulose LFA. The decimal logarithm of the *VR vs [cTnI]* data in Table S1 was taken and regression analysis was carried out with the regression function of Microsoft Excel (2010). The LOD and LOQ were computed using the standard deviation of response (σ) for the y intercept and slope of the log VR *vs* log [cTnI] calibration curve (see S1).

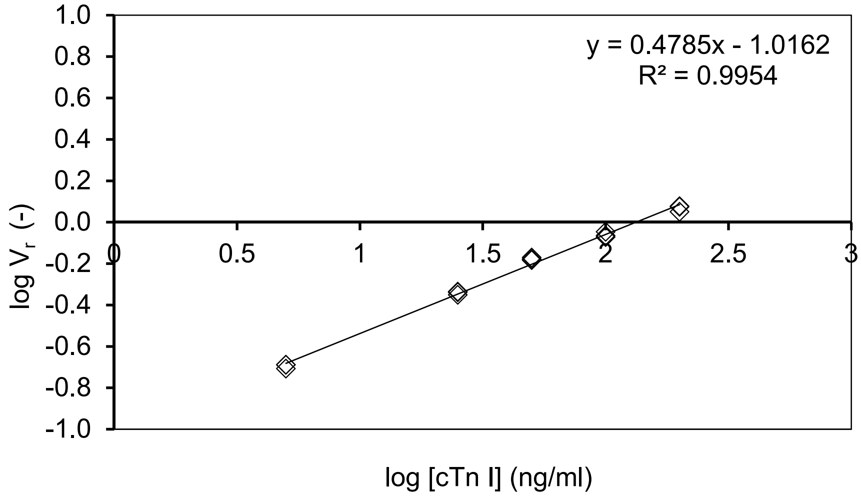

Slope	0.4785	LOD	1.28 ng/ml
Intercept	−1.0162	LOQ	2.10 ng/ml
Standard deviation (σ)	0.0154		

Figure S7. Linear regression of the log *VR vs* log *[cTnI]* data for the cellulose LFA. The decimal logarithm of the *VR vs [cTnI]* data in Table S2 was taken and regression analysis was carried out with the regression function of Microsoft Excel (2010). The LOD and LOQ were computed using the standard deviation of response (σ) for the y intercept and slope of the log VR *vs* log [cTnI] calibration curve (see S1).

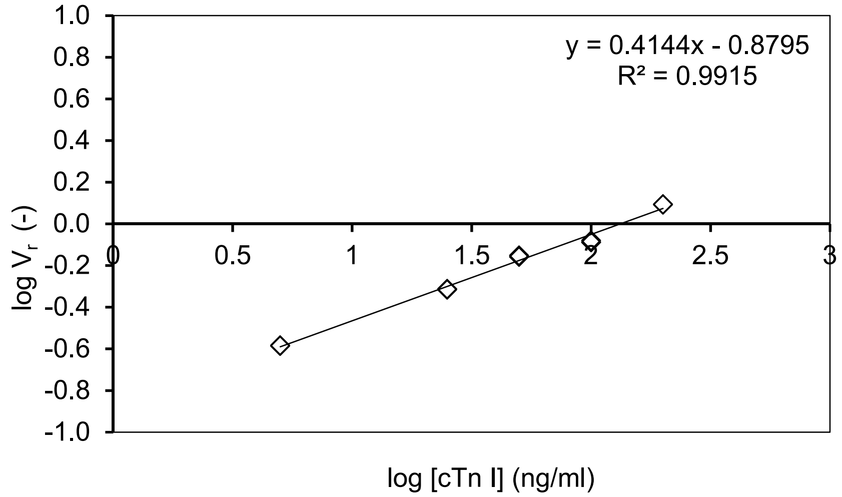

Slope	0.4144	LOD	1.40 ng/ml
Intercept	-0.8795	LOQ	2.75 ng/ml
Standard deviation (σ)	0.0182		

Figure S8. Linear regression of the log V_R *vs* log *[cTnI]* data for the cellulose CNF LFA. The decimal logarithm of the V_R *vs* *[cTnI]* data in **Table S3** was taken and regression analysis was carried out with the regression function of Microsoft Excel (2010). The LOD and LOQ were computed using the standard deviation of response (σ) for the y intercept and slope of the log V_R *vs* log [cTnI] calibration curve (see **S1**).

Table S1. Response of the nitrocellulose LFA to serum samples with different concentrations of cTnI and calculation of the corresponding intra-assay coefficient of variation. Experiments were performed in triplicate. Pixel volumes of the test (V_T) and control (V_C) lines, which are obtained from the fluorescence intensity data recorded by the ImageQuant analyser, are shown alongside with the corresponding mean volume ratio, V_R,.

[cTn I] (ng/ml)	V_T (-)	V_C (-)	V_R (-)	*SD* (-)	*Mean* (-)	*CoV* (%)
200	183864291	146545676	1.255			
200	183263018	144520046	1.268	0.0079	1.259	0.631
200	175748829	140148490	1.254			
100	122415708	145402574	0.842			
100	122375992	144963308	0.844	0.0014	0.843	0.161
100	123764423	146581339	0.844			
50	101693582	139005863	0.732			
50	101496787	140148490	0.724	0.0041	0.729	0.568
50	101300183	138546340	0.731			
25	74496120	149353694	0.499			
25	74507024	147926053	0.504	0.0026	0.501	0.515
25	74171408	148401173	0.500			
5	37905338	146581339	0.259			
5	37785479	145873510	0.259	0.0013	0.260	0.510
5	38145624	146109263	0.261			

Table S2. Response of the cellulose LFA to serum samples with different concentrations of cTnI and calculation of the corresponding intra-assay coefficient of variation. Experiments were performed in triplicate. Pixel volumes of the test (V_T) and control (V_C) lines, which are obtained from the fluorescence intensity data recorded by the ImageQuant analyser, are shown alongside with the corresponding mean volume ratio, V_R,.

[cTn I] (ng/ml)	V_T (-)	V_C (-)	V_R (-)	*SD* (-)	*Mean* (-)	*CoV* (%)
200	156866375	132263875	1.186			
200	157974426	132038841	1.196	0.0398	1.1684	3.407
200	151052684	134524755	1.123			
100	114475885	134979230	0.848			
100	114684878	133145917	0.861	0.0264	0.8695	3.041
100	114894061	127793869	0.899			
50	86008250	127312050	0.676			
50	86136466	131177272	0.657	0.0096	0.6650	1.450
50	86278997	130157878	0.663			
25	59034355	132298920	0.446			
25	59246636	128283336	0.462	0.0083	0.4556	1.820
25	59246636	129121589	0.459			
5	26149425	127573250	0.205			
5	26022222	132470907	0.196	0.0049	0.2021	2.434
5	26149425	127597091	0.205			

Table S3. Response of the cellulose-CNF LFA to serum samples with different concentrations of cTnI and calculation of the corresponding intra-assay coefficient of variation. Experiments were performed in triplicate. Pixel volumes of the test (V_T) and control (V_C) lines, which are obtained from the fluorescence intensity data recorded by the ImageQuant analyser, are shown alongside with the corresponding mean volume ratio, V_R,.

[cTn I] (ng/ml)	V_T (-)	V_C (-)	V_R (-)	*SD* (-)	*Mean* (-)	*CoV* (%)
200	189197279	151774560	1.247			
200	188392471	151619709	1.243	0.0052	1.242	0.416
200	188928819	152816807	1.236			
100	127208711	152501442	0.834			
100	126207590	154111548	0.819	0.0095	0.823	1.153
100	125895032	154151014	0.817			
50	108448019	153467403	0.707			
50	107637953	153306367	0.702	0.0058	0.701	0.833
50	107032396	153990104	0.695			
25	75001463	153626157	0.488			
25	74160174	153586754	0.483	0.0029	0.486	0.589
25	74664378	153223474	0.487			
5	40019020	152742647	0.262			
5	39648762	152501443	0.260	0.0013	0.261	0.480
5	39771990	153141533	0.260			

References

1. *Note for Guidance on Validation of Analytical Procedures: Text and Methodology (CPMP/ICH/381/95), ICH Topic Q 2 (R1)*; EMEA: London, UK, 2006.
